# Local and Regional Impacts of Pollution on Coral Reefs along the Thousand Islands North of the Megacity Jakarta, Indonesia

**DOI:** 10.1371/journal.pone.0138271

**Published:** 2015-09-17

**Authors:** Gunilla Baum, Hedi I. Januar, Sebastian C. A. Ferse, Andreas Kunzmann

**Affiliations:** 1 Department of Ecology, Leibniz Center for Tropical Marine Ecology (ZMT) Bremen GmbH, Bremen, Germany; 2 Faculty of Biology & Chemistry (FB2), University of Bremen, Bremen, Germany; 3 Indonesian Research Center for Marine and Fisheries Products Processing and Biotechnology, Slipi, Jakarta Pusat, Indonesia; National University of Singapore, SINGAPORE

## Abstract

Worldwide, coral reefs are challenged by multiple stressors due to growing urbanization, industrialization and coastal development. Coral reefs along the Thousand Islands off Jakarta, one of the largest megacities worldwide, have degraded dramatically over recent decades. The shift and decline in coral cover and composition has been extensively studied with a focus on large-scale gradients (i.e. regional drivers), however special focus on local drivers in shaping spatial community composition is still lacking. Here, the spatial impact of anthropogenic stressors on local and regional scales on coral reefs north of Jakarta was investigated. Results indicate that the direct impact of Jakarta is mainly restricted to inshore reefs, separating reefs in Jakarta Bay from reefs along the Thousand Islands further north. A spatial patchwork of differentially degraded reefs is present along the islands as a result of localized anthropogenic effects rather than regional gradients. Pollution is the main anthropogenic stressor, with over 80% of variation in benthic community composition driven by sedimentation rate, NO_2_, PO_4_ and Chlorophyll a. Thus, the spatial structure of reefs is directly related to intense anthropogenic pressure from local as well as regional sources. Therefore, improved spatial management that accounts for both local and regional stressors is needed for effective marine conservation.

## Introduction

Rising population numbers worldwide go hand in hand with an increase in the diversity and intensity of anthropogenic stressors. Coral reefs for instance are increasingly under pressure due to coastal development and resource use. At least 19% of reefs worldwide have been permanently lost [1], and of those remaining, over 60% are at immediate risk from direct human activities [2]. Local anthropogenic stressors can decouple reef communities from biophysical factors and become the principal drivers in shaping coral reef benthic community composition [3], leading to novel and unprecedented ecosystems that are composed and function in ways currently still poorly understood [4]. Therefore, understanding the processes that shape coral reef communities under the influence of multiple anthropogenic stressors is one of the core challenges in coral reef ecology and conservation. The most pertinent stressors on reefs have anthropogenic origins (e.g. nutrient input, fishing pressure or turbidity gradients due to coastal development [2]) and, depending on the specific location, occur at larger gradients or rather show localized impacts.

The degradation of reefs has direct impacts on coastal communities that depend on reef resources for their livelihoods. One of the main challenges of ecosystem and conservation management plans is to account for the connection between local habitats and the conflicting demands of different stakeholders on reef resources [5]. Marine spatial planning, which is based on assigning different activities to specific zones and thus can account for variable location-specific factors such as increasing distance from city centres or markets, has been proposed as an alternative to current non spatially-explicit management strategies. Such approaches may be especially suitable in areas with extreme urbanisation such as megacities, where numerous different stakeholders are involved in resource uses. However, there is still a lack in knowledge in how far the influence of large urban areas extends and to which extent local and regional anthropogenic and non-anthropogenic stressors interact.

Of today´s 28 megacities (cities with more than 10 million people), sixteen are located in Asia [6], and many megacities are located at the coast, causing various human-induced marine and coastal environmental problems such as water pollution, depletion of fishery resources, seafood contamination, loss of habitat, coastal littering as well as eutrophication and increased sedimentation rates [7].

The Greater Jakarta Metropolitan Area, as the 3rd largest agglomeration in the world with around 25 million inhabitants [8], and the Kepulauan Seribu (“Thousand Islands”) chain, located in front of Jakarta Bay (JB), represent an ideal area to assess the relative and interactive effects of multiple stressors on coral reef ecosystems. Here, local anthropogenic impacts have caused dramatic changes in coral reef ecosystems (e.g. [9]), especially over the past decades, with a current coral cover of < 5% for nearshore reefs within JB and < 20% cover in offshore reefs (up to 80 km north of the coast of Jakarta) (e.g. [10]). Coral reefs are of high economic and environmental importance for the Jakarta area and island communities, for instance supporting local fisheries, aquaculture and, to a lesser extent, a growing and mostly local tourism industry [2,11]. Currently, there are around 40,000 fishermen in JB and the Thousand Islands [12].

A marked inshore-offshore gradient in heavy metal pollution, nutrient input and water transparency [13,14], coral cover [15,16] and fish abundance [17] has been observed in the past. Cleary et al. [15] divided the island chain into three areas based on geomorphology, oceanography and distance from Jakarta, representing a disturbance gradient with severe pollution in nearshore reefs, medium pollution in midshore reefs and relatively minor anthropogenic disturbances in offshore reefs. However, localized stressors e.g. from destructive fishing and local nutrient sources are likely to be at least equally severe as regional impacts from coastal cities. This could potentially lead to high spatial variability in reef community composition and functioning, and previous research along the Thousand Islands has not addressed local drivers sufficiently. Furthermore, pollution from large cities and localized impacts from island communities require different management approaches.

Although the Thousand Islands constitute the oldest Marine National Park in Indonesia, management of the area is still ineffective [11]. Furthermore, comprehensive marine spatial management is lacking for the reefs outside the Thousand Island Marine National Park. This threatens not only local livelihoods, but also the stated goal of the Indonesian government to have 20 million hectares of marine area under effective management by 2020 [18]. In order to develop successful management and conservation strategies for the coral reefs in JB and the Thousand Islands, the spatial scale at which stressors act has to be determined. Thus, the present study assessed whether spatial trends in stressors and benthic community composition reflect the distance to Jakarta, reef conditions are a reflection of localized effects, or (and to what extent) a combination of both regional and local effects is shaping local reef communities. In addition, it was examined which of the different stressors mainly shape the local structure of coral reefs along the island chain.

## Material and Methods

### Study area

The Kepulauan Seribu (Thousand Islands) are comprised of 105 small (< 10 ha) and very low-lying (< 3 m above sea level) islands, most with lagoons and fringing reefs, reaching up to 80 km north of the city [19]. In 1982 Indonesia´s first Marine National Park, the Thousand Islands National Park, was established in the north of the island chain [20]. Reef development generally is restricted to shallow depths (around 3–10 m, max. 20 m depth). Although reefs within the bay once had thriving coral communities [21,22], they are now dominated by sand, rubble and algae. Several islands within the bay were mined for construction purposes and have eroded away [23–25]. With a total population of around 22,700 people, the island chain is densely populated. 65% of the people live on the four main islands Panggang, Pramuka, Kelapa and Harapan [12]. Numerous stakeholders are presently involved in fishing, sand mining, tourism and aquaculture, in particular the culture of green mussels (*Perna viridis*) in JB. The latter has a daily production of around 20–25 t and involves around 3,000 fisher families [19]. Several rivers with a combined catchment area of 2000 km^2^ discharge directly into the bay and transport large amounts of untreated sewage and industrial effluent with high pollutant levels [13]. Around 60% of the bay´s shoreline has been modified due to massive urbanization and industrialization as well infrastructural development in Jakarta, and another 30% for agricultural or aquaculture developments [26]. During the dry season, the predominantly south-easterly winds can cause polluted surface waters from the JB area to reach midshore reefs, while during the wet season, north-westerly winds blow from offshore towards JB [15].

**Fig 1:**
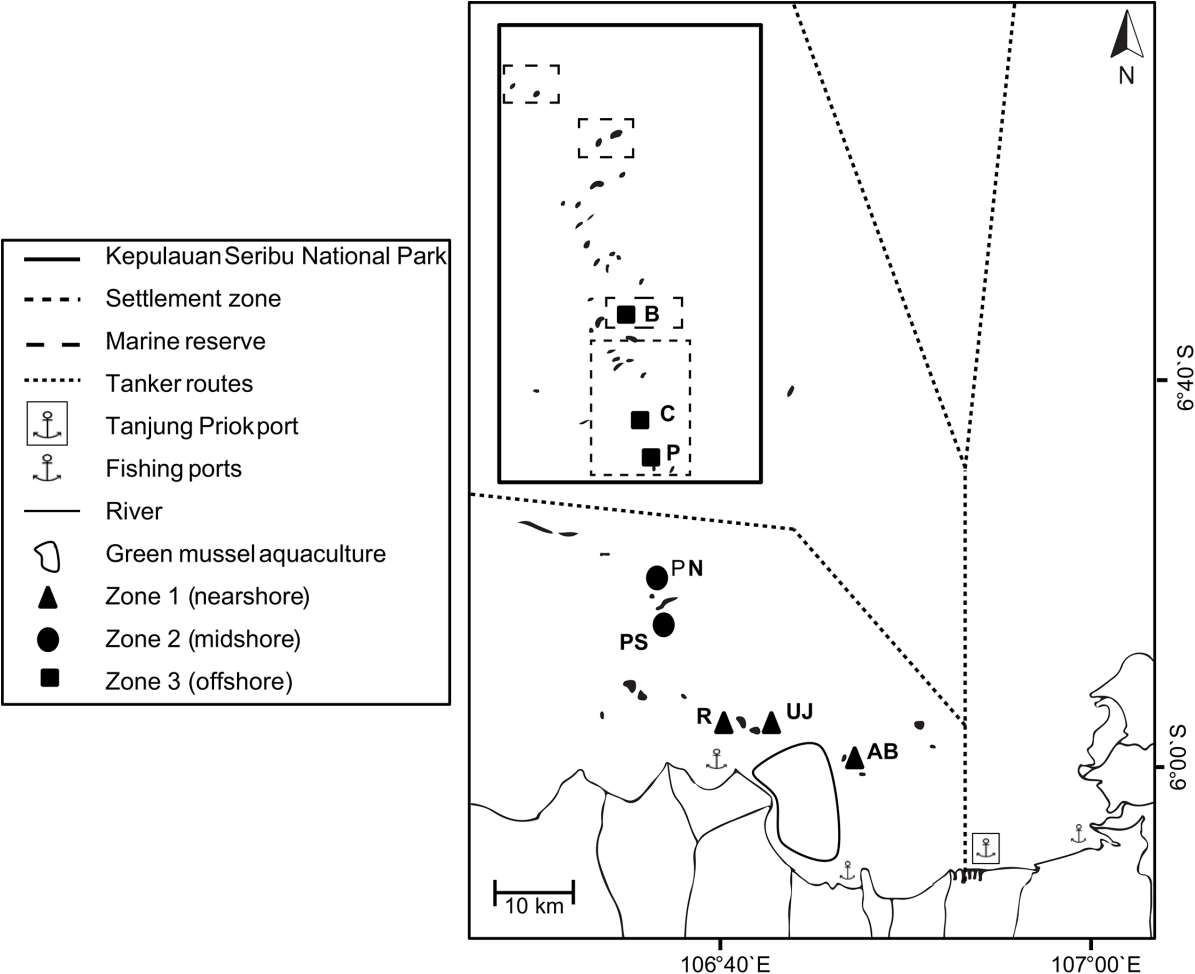
Study area. Map includes boundaries of the Thousand Islands Marine National Park, ports and study sites from nearshore reefs (within Jakarta Bay) as well as from the outer Thousand Islands (mid- and offshore): AB = Ayer Besar, UJ = Untung Jawa, R = Rambut, PS = Pari South, PN = Pari North, P = Panggang, C = Congkak, B = Bira.

For this project, eight coral reef sites across the Thousand Islands chain were visited in November 2012 during the transition time between northwest and southeast monsoon. The field studies did not involve endangered or protected species. Sites within JB (nearshore zone; < 20km) as well as from the outer Thousand Islands (mid- and offshore zones; 20–45 km and > 45 km, respectively) were chosen to represent both inhabited and non-inhabited islands for each of the three zones. Following the same methodology as previous studies [15,27], reefs from the northern side of each island were visited to ensure consistent wave exposure and current regimes. In case strong waves did not allow anchoring on the northern side of islands, transects were placed slightly to the north-east (Kayu Angin Bira (B) and Gosong Conkak (C)). An exceptional case was Pari: here, the south side of the island was included to account for previously observed strong differences in coral cover between the northern and southern side of the island [28,29] (Table 1, Fig 1).

**Table 1:** Description of sampling sites.

Site	Site abbrev.	Zone	Coordinate [E]	Coordinate [S]	Linear distance to Jakarta [km]	Permanent residents	Characteristics
Ayer Besar	AB	1	106°42.242	05°58.399	11.3	-	Private, for tourism
Untung Jawa	UJ	1	106°46.911	05°58.399	16.4	1726	Small settlement
Rambut	R	1	106°41.597	05°58.202	17.3	-	No permanent settlement
Pari South	PS	2	106°36.963	05°52.094	31.4	2458	Large fringing reef, tourism, research station
Pari North	PN	2	106°37.440	05°51.001	32.6		Large fringing reef, tourism, research station
Gosong Panggang	P	3	106°35.355	05°44.664	45.7	5123	Coral key, close to very densely populated islands (Pramuka and Panggang)
Gosong Conkak	C	3	106 35.274	05 42.303	49.5	-	Coral key
Kayu Angin Bira	B	3	106°34.162	05°36.405	59.8	-	Within conservation area of the NP, i.e. no fishing activities

Description of sampling sites (linear distance refers to distance from each site to the port Muara Angke in Jakarta). Zonation is based on Tomascik et al. [30] and DeVantier et al. [25]: zone 1 (nearshore; within Jakarta Bay): < 20 km; zone 2 (midshore): 20–45 km; zone 3 (offshore): > 45 km. Number of residents is given per `administrative village`(Indonesian: *kelurahan*) that contain several islands within the island complex Untung Jawa, Pari and Panggang.

### Benthic and reef fish community

Benthic habitat structure was assessed at each location using three replicate 50 m line-intercept transects at 5 +/- 0.5 m water depth [31]. Preliminary visits to the Thousand Islands had shown that highest coral cover can be commonly found at shallower depth and that at nearshore sites turbidity was too high at greater depth to conduct accurate surveys. Therefore a depth of 5 m was chosen to allow for adequate comparison across the sites which would not be possible at greater depths. High-resolution underwater photographs were taken using a digital camera (Canon G12 in a WP-DC 28 housing) every two meters on both sides of the transect line with a 1x1 m gridded quadrat frame for reference. All three replicate transects at each site were conducted on the same day between 8:00 h and 13:00 h. For the assessment of benthic community composition, photographs were analysed using CPCE software [32]. 50 random points were placed on each photo and each point was assigned to one the following benthic categories: hard and soft corals, acroporid and non-acroporid corals, recently dead corals (coral structure largely intact and attached to sediment), sand, pavement and rubble (bare or overgrown with turf or crustose coralline algae; rubble objects can move during storms/currents) and macroalgae. Overall total live coral cover was calculated. In addition, corals were further separated into morphological categories [33,34] to assess coral morphology composition. The relative abundance of these standardized coral morphology categories was used to classify coral reefs from JB and the Thousand Islands using a r-K-S- ternary (triangular) plot (based on [34], see S3 Table and S1 Fig).

Fish community composition was assessed along the same transects as benthic habitat structure using underwater visual census [31]. Number of fish species and number of individuals per species was recorded within 2.5 m of each side of the transect line (yielding a total area of 250 m^2^ per transect) by slowly swimming along the line at a constant speed. Fish surveys were always conducted by the same person at all sites to minimize observer bias. Each fish species was further assigned to one of five different feeding guilds to gain information on feeding guild composition (based on information from FishBase [35]: herbivores (HV), carnivores (CV), planktivores (PV), omnivores/invertivores (OVI) and obligate corallivores (OCV).

### Water quality

At each sampling site, temperature (°C), dissolved oxygen (DO; mg/L), pH, salinity (PSU), turbidity (NTU) and Chl a (μg/L) concentration of the water were measured as common water quality indicators (e.g. [36,37]) at 1 and 3 m water depth using a Eureka 2 Manta Multiprobe (Eureka Environmental Engineering, Texas, USA). Measuring interval was set to 1 min. Measurements of 3–4 min duration were taken twice a day, once in the morning around 09:00 h and once in the afternoon around 13:00 h. Water samples for inorganic nutrient analysis (nitrite (NO_2_), nitrate (NO_3_), phosphate (PO_4_), ammonia (NH_3_)) were taken at each sampling site at 1 and 4.5 m water depth. Samples were stored in an ice cooler and analysed the same day using a field photometer. Dissolved inorganic nitrogen (DIN) is given as the sum of NO_2_, NO_3_ and NH_3_.

Sediment traps made from a PVC tube with a height-to-width ratio of 7.2 (as recommended by Storlazzi et al. [38]) were deployed at 5 +/- 0.5 m depth for 22 +/- 1 h at each location (n = 4 per site). Traps were sealed underwater prior to retrieval. All water and sediment in the tubes was transferred to plastic bottles (5 L) and samples were stored in the dark until further processing. Water was filtered through Whatman GF/C glass microfiber filters (diameter 110 mm; 1.2 μm porosity) that had been precombusted at 500°C for 6 h and weighed. After filtering, filters were dried at 65°C for 24 h and re-weighed. Sedimentation rate is given as total particulate mass flux (TPMF) [g m^-2^ d^-1^] according to UNESCO [39]:

$$TPMF=\frac{DW}{A_{r}T}$$

where DW is dry weight of trapped sediment samples [g], T is trapping duration [d] and A_r_ is the area of the sediment trap tube opening [m^2^] with π = 3.14 and d = aperture size [cm]:

$$A_{r}=\pi {\left(0.5d\right)}^{2}10^{-4}$$

### Statistical analysis

Spatial trends for each individual water and biological factor along the islands in JB and the Thousand Islands were analyzed for effects of the distance to Jakarta and localized patterns among sites. To test whether the large-scale effects emanating from Jakarta act linearly or non-linearly along the distance gradient, a number of different models were used in assessing large-scale trends: grouping of sites into *a priori* defined zones (blocked treatments: near-, mid- and offshore), gradual in- or decreases (linear regression), exponential in- or decreases (exponential regression), and linear regression with one breakpoint (i.e. two linear segments). Differences in water and biological factors among zones and sites were analysed using one-way ANOVA. Data were checked for assumptions of normality and homogeneity of variances. In case assumptions were not fulfilled, a Kruskal Wallis test was performed instead. If significant effects were detected, pairwise comparisons with the post-hoc Tukey test were performed to assess significant differences between individual factors. Univariate statistics were performed with SigmaPlot 12.5.

Multivariate statistics of spatial trends for each of the four different biological composition groups (fish community, fish feeding guild, benthic community and coral morphology) were performed using PRIMER-E software v.6 [40]. In order to account for different scales and units [41], the water factors PO_4_, NH_4_, NO_3_, turbidity and Chl a were log+1 transformed, followed by normalization of all water factors. All biological factors were square root transformed prior to further analysis in order to reduce the influence of overly abundant species [42]. Bray-Curtis similarity matrices [43] were calculated for all biological composition groups and Euclidian distance was used to construct the similarity matrix for water data [40]. Permutational multivariate analysis of variance (PERMANOVA) was used to test for significant differences among *a priori*-defined groups (i.e. three zones) [44]. Distance-based redundancy analysis (dbRDA; [44]), a constrained ordination technique, was used to visualise differences between zones. Furthermore, the role of individual stressors was assessed with the BEST routine (using the BioEnv procedure based on Spearman rank correlation; [45]) to determine which of the factors best explained the composition of fish and benthic composition groups. Only proximate drivers were considered here: 1) water factors for both benthic composition groups and 2) benthic community and coral morphology factors for fish composition groups respectively. Those factors identified by BEST were then used for the LINKTREE procedure [45] in PRIMER to construct a linkage tree, a hierarchical tree that shows how fish and benthic compositions separate into zones. The RELATE test, a comparative (mantel-type) test on similarity matrices, was used to compare the different composition groups (Spearman rank correlation; Rho = 1 indicates a perfect match; [46]).

## Results

### Spatial trends by factors

**Fig 2:**
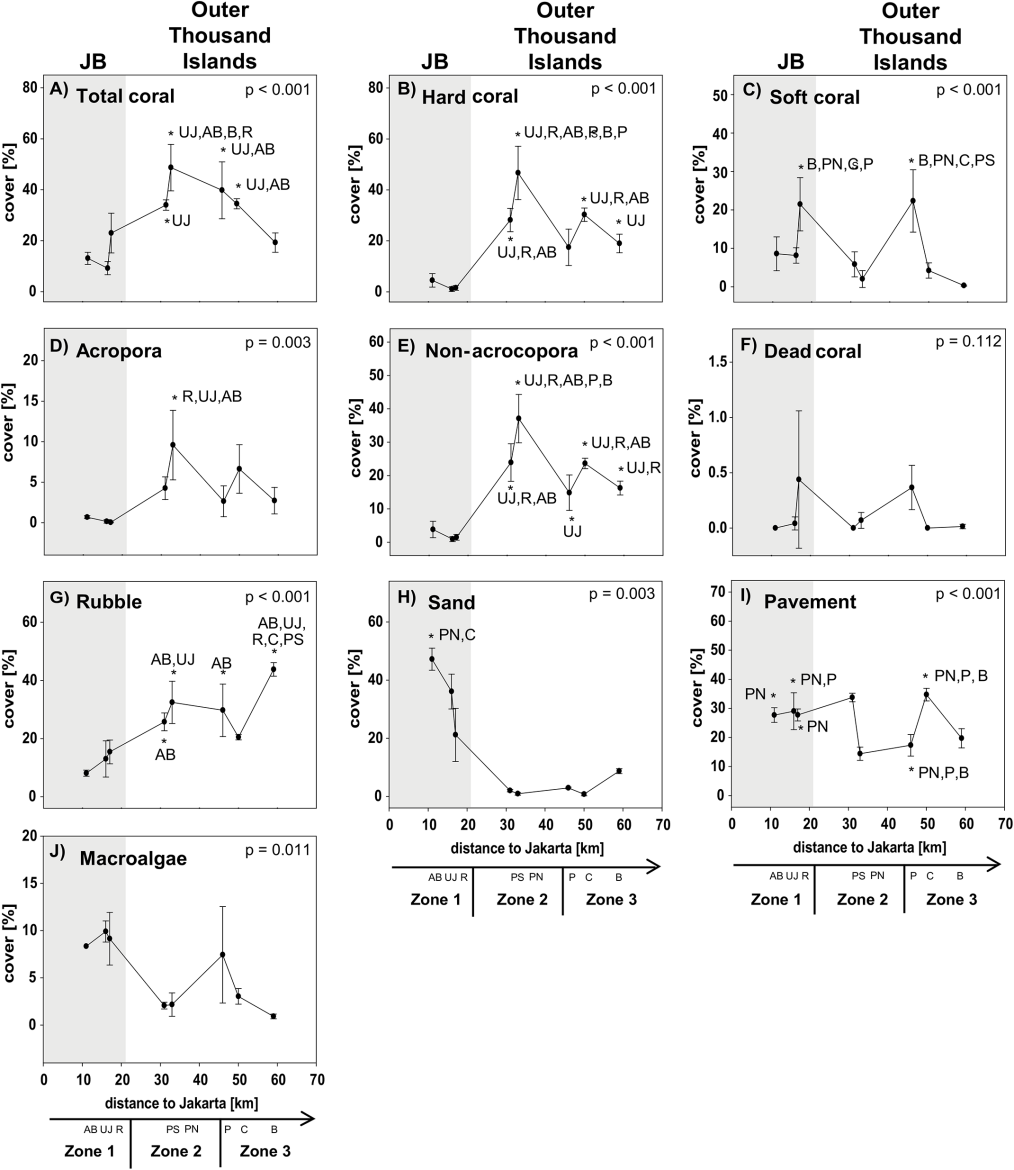
Benthic community composition. Data for sites in Jakarta Bay (JB; grey highlighted) and outer Thousand Islands is given as mean cover (± SD) for total coral (A), hard coral (B), soft coral (C), acroporid (D), non-acroporid (E), dead coral (F), rubble (G), sand (H), pavement (I) and macroalgae (J) at each site. p-values and post hoc results for differences between sites are given for each graph. Consider different scales on y-axis. Study sites: AB = Ayer Besar, UJ = Untung Jawa, R = Rambut, PS = Pari South, PN = Pari North, P = Panggang, C = Congkak, B = Bira.

A total of 22 families and 92 species of fishes were observed overall (S1 Table). Fish abundance at each site was dominated by the family Pomacentridae, encompassing > 60% at each site, followed by Labridae with around 15%. Within inshore reefs, total fish abundance was extremely low (65 ± 27.8 ind. 250 m^-2^, mean ± SD), while in midshore (354 ± 72.4 ind. 250 m^-2^, mean ± SD) and offshore reefs (335 ± 127.3 ind. 250 m^-2^, mean ± SD) abundances were very similar. Species richness 250 m^-2^ was lowest in inshore (11 ± 4.1, mean ± SD) and similar in mid- (27 ± 5.9, mean ± SD) and offshore reefs (21 ± 2.1, mean ± SD). The highest fish diversity (Shannon biodiversity index) was found at Pari North in the midshore zone. Sites within JB (inshore) did not differ from offshore sites with regard to fish diversity (Table 2). Highest total coral cover of 49% (mean hard coral cover: 47%) was found at Pari North in the midshore zone, while near- and offshore reefs had a total coral cover of 15 ± 9.1% (mean ± SD; the majority of which were soft corals (mainly nephtheids, xeniids and alcyoniidids); hard coral cover was 2 ± 2.3% (mean ± SD) and 31 ± 11.1% (mean ± SD; hard coral cover: 22 ± 7.5%, mean ± SD), respectively (Fig 2).

**Table 2:** Diversity indices for fish community.

Site	Total species S	Total individuals N	Shannon H´(log)	Simpson 1-λ`
**AB**	20	58	2.54	0.90
**UJ**	27	99	2.82	0.92
**R**	18	38	2.72	0.95
**PS**	38	354	2.96	0.93
**PN**	48	353	3.10	0.94
**P**	37	300	2.67	0.88
**C**	35	354	2.81	0.92
**B**	30	349	2.66	0.90

Fish diversity (Shannon H´), evenness (Simpson 1- λ), total number of species (S) and individuals (N) for each site respectively.

**Fig 3:**
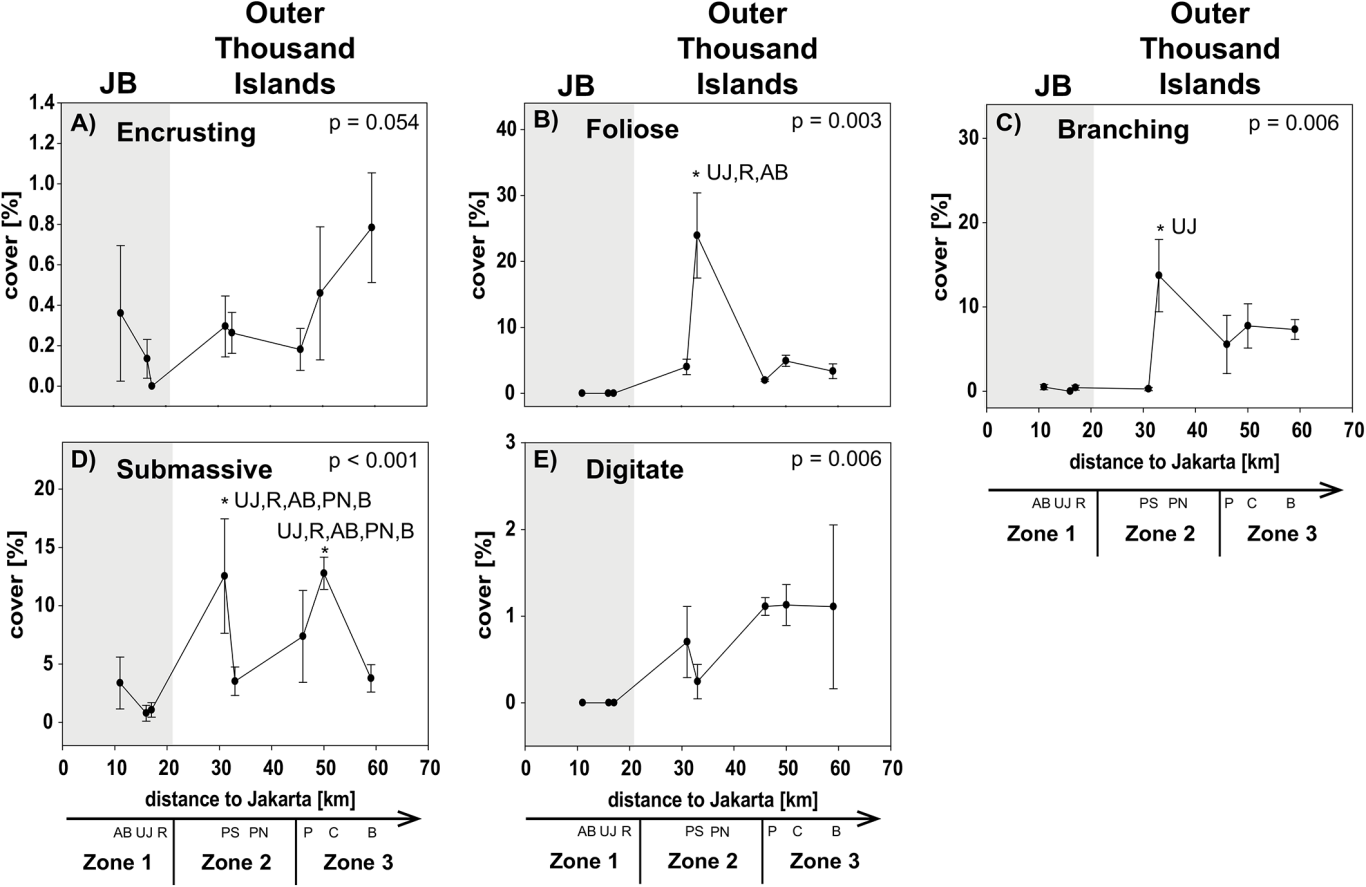
Coral morphology composition. Data for sites in Jakarta Bay (JB; grey highlighted) and outer Thousand Islands is given as mean cover (± SD) for encrusting (A), foliose (B), branching (C), submassive (D) and digitate (E) corals at each site. p-values and post hoc results for differences between sites are given for each graph. Consider different scales on y-axis. Study sites: AB = Ayer Besar, UJ = Untung Jawa, R = Rambut, PS = Pari South, PN = Pari North, P = Panggang, C = Congkak, B = Bira.

Inshore, corals were comprised almost exclusively of submassive corals which are stress-tolerant corals (S) [47,48] and encrusting corals, which are competition-adapted corals (K), while foliose, digitate and branching corals were completely or almost absent (Fig 3). The two sites Pari North and Bira were dominated by competition-adapted corals (K), i.e. all non-acroporid corals that are either branching, encrusting, foliose or mushroom-shaped and are characterized as K-adapted due to their lower growth rates than acroporid corals [49,50]. Ruderal corals however (r), i.e. all acroporid corals and tabular non-acroporid corals, which are more disturbance-adapted due to their rapid growth and mechanical fragility [51,52], were not dominating any reefs (Fig 3 and S1 Fig).

**Fig 4:**
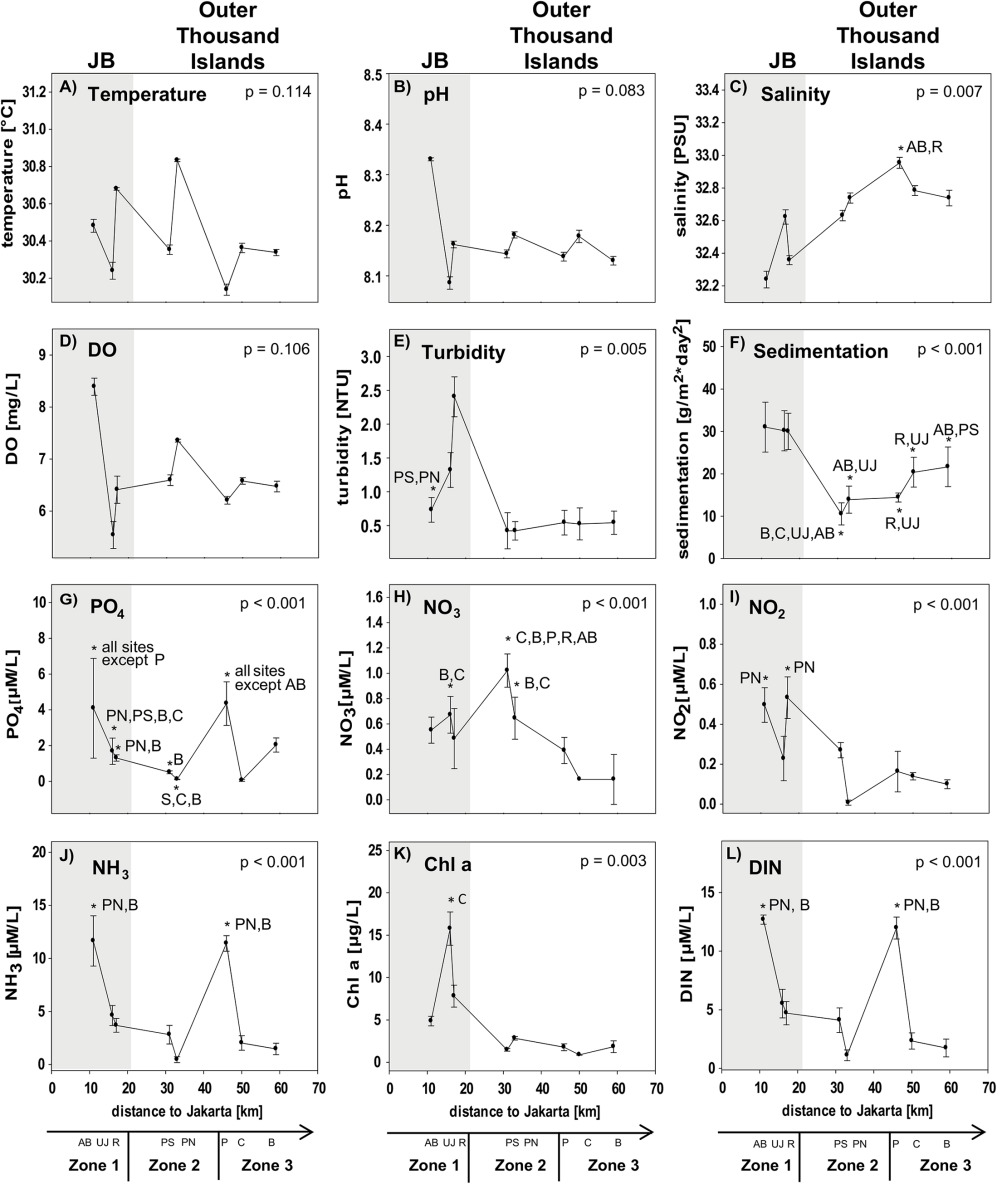
Water quality composition. Data for sites in Jakarta Bay (JB; grey highlighted) and outer Thousand Islands: Mean values (± SD) for the factors (A) temperature [°C], (B) pH, (C) salinity [PSU], (D) DO [mg/L], (E) turbidity [NTU], (F) sedimentation, the inorganic nutrients [μM/L] PO_4_ (G), NO_3_ (H), NO_2_ (I) as well as NH_3_ (J), (K) Chl a [μg/L] and (L) dissolved inorganic nutrients (DIN; [μM/L] at each site. p-values and post hoc results for differences between sites are given for each graph. Study sites: AB = Ayer Besar, UJ = Untung Jawa, R = Rambut, PS = Pari South, PN = Pari North, P = Panggang, C = Congkak, B = Bira.

**Fig 5:**
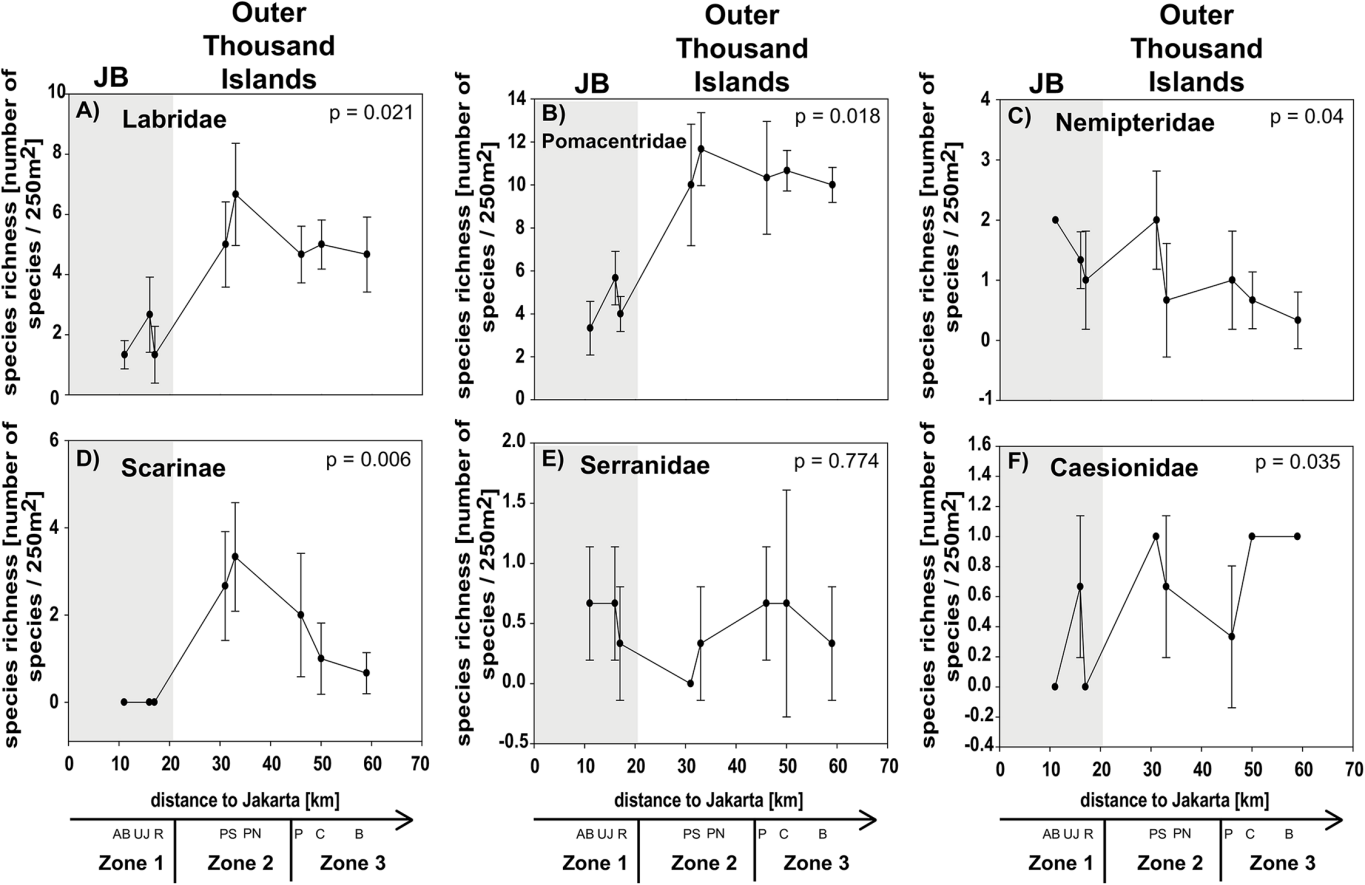
Fish community composition: Species richness. Data for sites in Jakarta Bay (JB; grey highlighted) and outer Thousand Islands is given as mean species richness (± SD) for the families (A) Labridae (excluding Scarinae), (B) Pomacentridae, (C) Nemipteridae, (D) labrid Scarinae, (E) Serrranidae and (F) Caesionidae at each site. p-values and post hoc results for differences between sites are given for each graph. Consider different scales on y-axis. Study sites: AB = Ayer Besar, UJ = Untung Jawa, R = Rambut, PS = Pari South, PN = Pari North, P = Panggang, C = Congkak, B = Bira.

**Fig 6:**
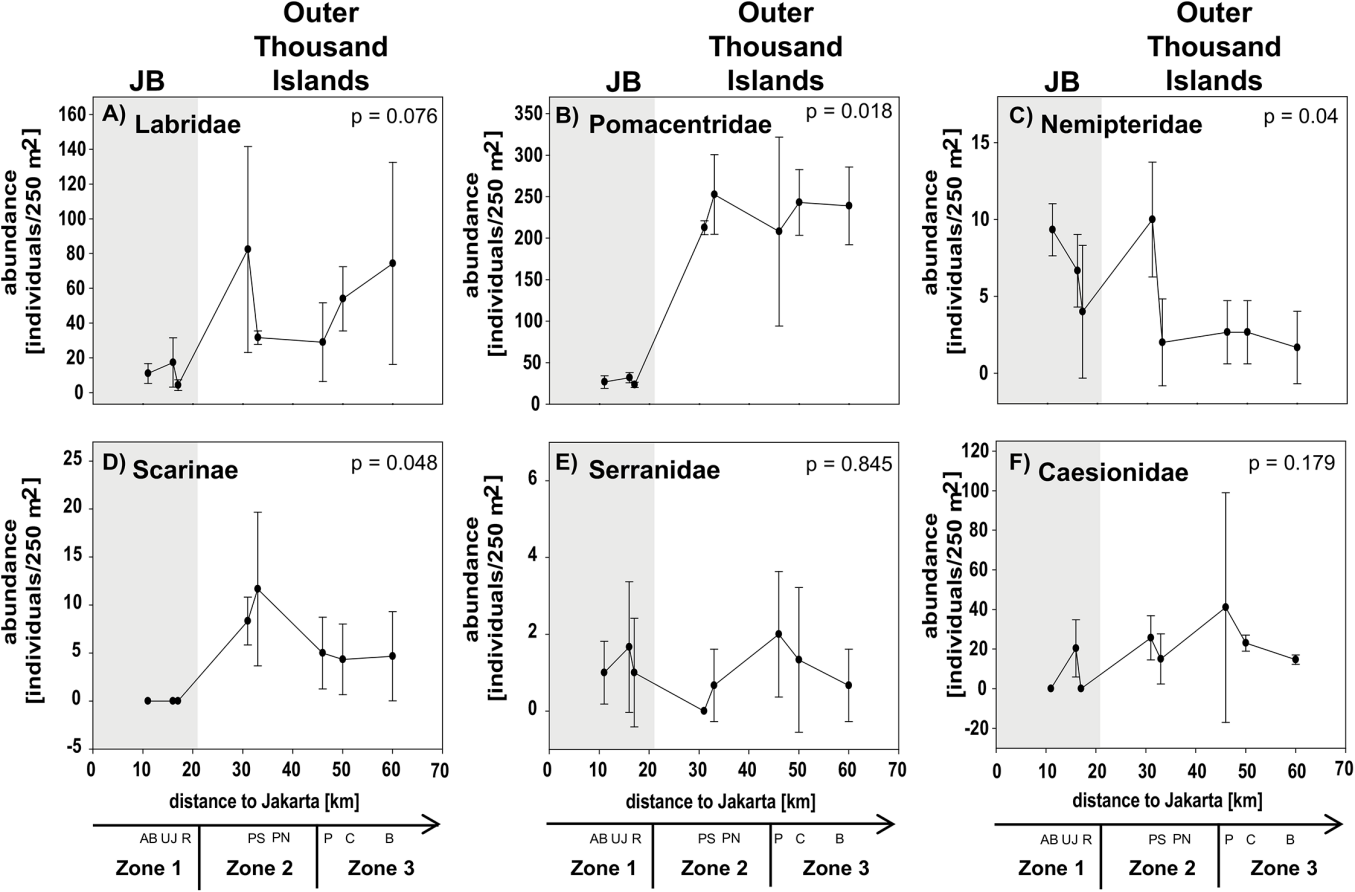
Fish community composition: Fish abundance. Data for sites in Jakarta Bay (JB; grey highlighted) and outer Thousand Islands is given as mean values (± SD) for the families (A) Labridae (excluding Scarinae), (B) Pomacentridae, (C) Nemipteridae, (D) labrid Scarinae, (E) Serrranidae and (F) Caesionidae at each site. p-values and post hoc results for differences between sites are given for each graph. Note the different scales on y-axis. Study sites: AB = Ayer Besar, UJ = Untung Jawa, R = Rambut, PS = Pari South, PN = Pari North, P = Panggang, C = Congkak, B = Bira.

**Fig 7:**
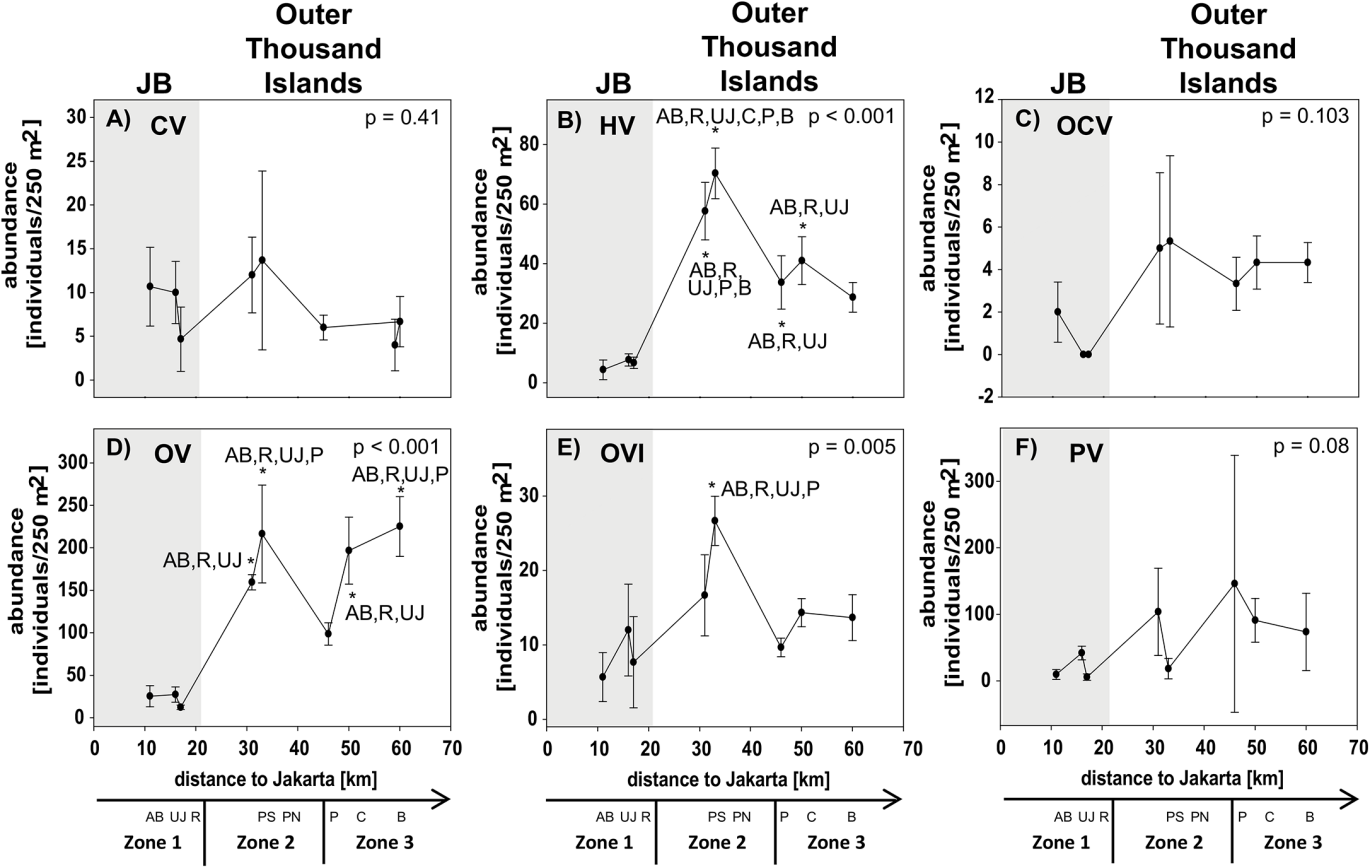
Fish feeding guild composition. Data for sites in Jakarta Bay (JB; grey highlighted) and outer Thousand Island is given as mean abundance (± SD) for the guilds (A) corallivores (CV), (B) herbivores (HV), (C) obligate corallivores (OCV), (D) omnivores (OV), (E) omnivore/invertivores (OVI) and (F) planktivores (PV) at each site. p-values and post hoc results for differences between sites are given for each graph. Consider different scales on y-axis. Study sites: AB = Ayer Besar, UJ = Untung Jawa, R = Rambut, PS = Pari South, PN = Pari North, P = Panggang, C = Congkak, B = Bira.

A clear separation of all three zones was only found for the feeding guild OV and for the species richness in the subfamily Scarinae. The separation of mid- and offshores sites within the Thousand Islands was less distinct. Only some factors showed a significant separation of inshore sites and sites from the Thousand Islands (e.g. NO_2_, cover of hard and non-acroporid corals, total fish abundance and species richness, abundance of Pomacentridae and Scarinae, species richness of Pomacentriade and Caesionidae; see Table 3 for post-hoc results; Figs 4–7).

**Table 3:** Univariate analyses to test for distance-based and localized spatial trends for 5 different fish and benthic community composition groups and environmental factors.

Factors			Distance-based effects					Localized effects
Group	Composition	Component/Factor	Regression analysis—best fit			Zonation (*a priori* groups)		p-value
			Type	p-value	R^2^	p-value	Post-hoc	
Fish	Community: abundance	Caesionidiae	Linear	0.16	0.3	0.196		0.179
		Pomacentridae	Linear (2 segments)	**< 0.001**	0.95	**0.002**	z1 vs. z2 z1 vs.z3	**0.018**
		Labridae	Linear	**0.05**	0.49	0.086		0.079
		Nemipteridae	Exp.	0.08	0.43	**0.04**		0.11
		Scaridae	Linear (2 segments)	**0.03**	0.86	**0.03**	z1 vs. z2 z1 vs.z3	**0.048**
		Serranidae	Exp.	0.23	0.23	0.08		0.845
		Total	Linear (2 segments)	**0.01**	0.93	**< 0.001**	z1 vs. z2 z1 vs.z3	**0.014**
	Community: species richness	Caesionidiae	Linear	0.08	0.44	**0.033**	z1 vs. z2 z1 vs.z3	**0.035**
		Pomacentridae	Exp.	**< 0.001**	0.79	**0.026**	Z1 vs. z3	**0.018**
		Labridae	Linear (2 segments)	**0.02**	0.9	**0.002**	z1 vs. z2 z1 vs.z3	**0.021**
		Nemipteridae	Linear	0.06	0.48	0.261		0.204
		Scaridae	Linear (2 segments)	**0.01**	0.92	**< 0.001**	z1 vs. z2 z1 vs.z3 z2 vs.z3	**0.006**
		Serranidae	Linear (2 segments)	0.58	0.36	0.148		0.774
		Total	Linear (2 segments)	**0.04**	0.86	**0.006**	z1 vs. z2 z1 vs.z3	**< 0.001**
	Feeding guild	CV	Linear (2 segments)	0.25	0.61	0.445		0.41
		HV	Linear (2 segments)	**0.01**	0.92	**< 0.001**	z1 vs. z2 z1 vs.z3	**< 0.001**
		OV	Linear	**0.02**	0.66	**< 0.001**	z1 vs. z2 z1 vs.z3 z2 vs.z3	**< 0.001**
		OVI	Linear (2 segments)	0.21	0.65	0.382		**0.005**
		PV	Linear	0.07	0.45	0.159		0.08
		OCV	Exp.	**0.05**	0.49	0.095		0.103
Benthic	Coral morphology	Digitate	Linear	**< 0.001**	0.88	**0.011**		**0.006**
		Submassive	Linear (2 segments)	0.41	0.48	0.213		**< 0.001**
		Branching	Linear	0.11	0.37	**0.006**		0.286
		Foliose	Linear (2 segments)	0.41	0.48	**0.025**		**0.003**
		Encrusting	Linear	**0.06**	0.48	0.368		0.054
	Community	Dead coral	Exp.	0.81	0.01	0.798		0.112
		Hard coral	Linear	0.06	0.67	**0.009**	z1 vs. z2 z1 vs.z3	**< 0.001**
		Rubble	Exp.	**0.01**	0.71	0.067		**< 0.001**
		Pavement	Linear	0.57	0.08	0.808		**< 0.001**
		Sand	Linear (2 segments)	**0.01**	0.94	0.061		**0.003**
		Macroalgae	Linear	0.47	0.09	0.543		**0.011**
		Soft coral	Linear	0.48	0.09	0.591		**< 0.001**
		Acroporid	Linear (2 segments)	0.19	0.66	0.054		**0.003**
		Non-acroporid	Linear (2 segments)	0.07	0.8	**0.005**	z1 vs. z2 z1 vs.z3	**< 0.001**
		Total coral	Linear (2 segments)	**0.03**	0.87	0.062		**< 0.001**
Water		Salinity	Linear	**0.02**	0.6	**0.049**	z1 vs. z3	**0.007**
		pH	Exp.	0.37	0.13	0.806		0.083
		DO	Exp.	0.56	0.06	0.837		0.106
		Temperature	Linear	0.44	0.1	0.357		0.114
		Turbidity	Exp.	0.15	0.32	**0.011**	z1 vs. z2	**0.005**
		Sedimentation	Linear (2 segments)	**0.01**	0.92	0.244		**< 0.001**
		Chl a	Exp.	0.07	0.37	0.081		**0.003**
		PO_4_	Exp.	0.7	0.03	0.421		**< 0.001**
		NO_3_ (nitrate)	Linear	0.42	0.11	**0.025**		**< 0.001**
		NO_2_ (nitrite)	Linear	**0.03**	0.57	0.088		**< 0.001**
		NH_4_	Exp.	0.47	0.54	0.524		**< 0.001**
		DIN	Linear	0.15	0.35	0.54		**< 0.001**

Regression types tested were: linear or exponential de- or increase towards offshore and linear de- or increase with one breakpoint (2 segments). The regression type yielding the lowest p-value and highest R^2^ is shown. Tests for zonation and localized effects were done using one-way Anova. Post-hoc results (Tukey test) for differences between zones are given in the ‘zonation’ column.

Among the different factors of each composition group, only few showed significant gradual or exponential in- or decreases with increasing distance from shore. Clear linear increases in salinity and cover of digitate corals and a decrease in NO_2_ as well as exponential increases in coral rubble were found towards offshore. Other factors first decreased significantly from Ayer Besar to Rambut in JB and then either increased linearly (e.g. sand, sedimentation rate, total fish species richness) or decreased (e.g. abundance of Pomacentridae, herbivores; see Table 3, Figs 4–7).

Most factors did not show a clear zonation or distance-based spatial patterns, but rather localized patterns. The concentration of PO_4_ and NH_3_ for example both seemed to decrease towards offshore, however both showed a significantly higher concentration at a single offshore site (Panggang) compared to all other sites. Also at Bira, a site that is furthest north and within the conservation zone of the National Park, PO_4_ was higher than at midshore sites, where concentrations were lowest overall. Similarly, such a local but significantly higher increase was found at Pari South for NO_3_ concentrations and for Chl a at Untung Jawa. Also soft coral cover had two peaks at Rambut and Panggang, where cover was significantly higher than at Pari North and Bira. At Pari North coral cover (hard, acroporid, non-acroporid and foliose) was higher compared to all nearshore sites and to several offshore sites (Table 3, Figs 4–7).

### Relative role of stressors and interactions between composition groups

**Fig 8:**
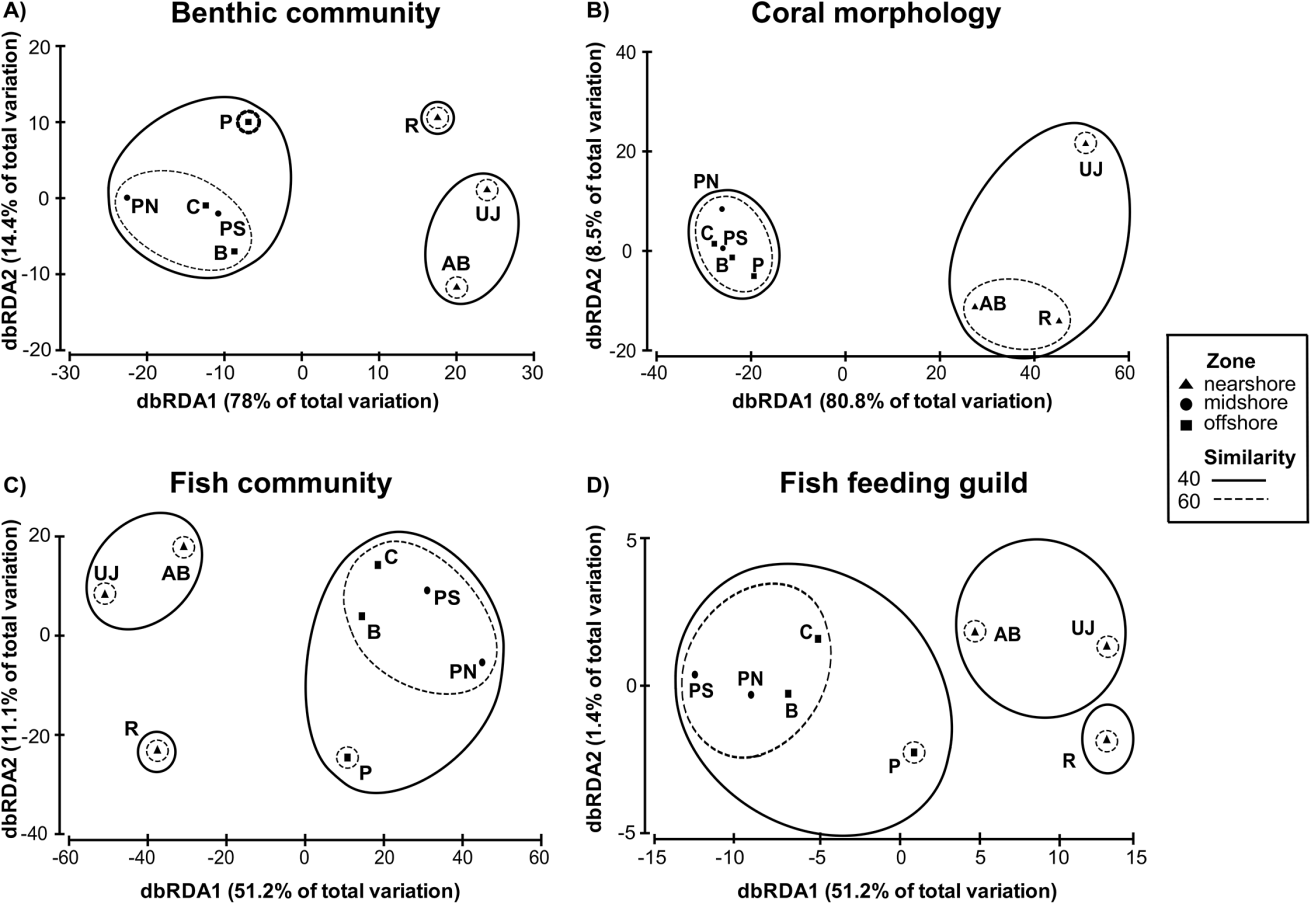
Visualization of fish and benthic community composition based on distance-based redundancy analysis (dbRDA). Benthic community composition (A), coral morphology composition (B), fish community taxonomic composition (C) and fish feeding guild composition (D) are shown. Study sites: AB = Ayer Besar, UJ = Untung Jawa, R = Rambut, PS = Pari South, PN = Pari North, P = Panggang, C = Congkak, B = Bira.

Multivariate analysis of each composition group with a zonal distribution pattern as basis revealed an overall significant difference between zones for all composition groups (Permanova test; p < 0.05), although pairwise tests for effects of zones failed to detect significant differences (p > 0.05). The results indicate that differences were mainly between the first and one of the other two zones (S2 Table, S2 Fig). Similar to results from the univariate analysis, the separation of nearshore sites in JB and offshore sites was distinct (Fig 8). Offshore sites however clustered together and displayed no clear separation into mid- and offshore zones. For both fish composition groups and the benthic community composition, the site Panggang was less similar to the other offshore sites from the Thousand Islands (Fig 8).

Benthic community (p = 0.007) and coral morphology (p = 0.016) composition were significantly related to water characteristics (Table 4). Spatial patterns in the benthic community composition were best explained by NO_2_ concentration, followed by sedimentation rate, PO_4_ and Chl a, together accounting for 83% of the observed variability. Coral morphology composition was explained by NO_2_, turbidity, sedimentation and Chl a, which accounted for 88% of the observed variability (Table 5).

**Table 4:** Relation (test: RELATE) between composition groups and their proximate driving composition group.

Group	Composition	Relation with		
		Composition (proximate driver)	R	p-value
Benthic	Community	Water	0.61	**0.007**
	Coral morphology	Water	0.59	**0.016**
Fish	Community	Benthic community	0.8	**0.003**
		Coral morphology	0.88	**0.002**
	Feeding guild	Benthic community	0.58	**0.013**
		Coral morphology	0.78	**0.003**

**Table 5:** Correlation of each composition group with their proximate driving composition group.

		Correlation with				
Group	Composition	Composition	All Thousand Islands		Outer Thousand Islands	
			Corr	Factor	Corr	Factor
Benthic	Community	Water	0.83	Chl a	0.69	PO_4_
				NO_2_		Sal
				PO_4_		DO
				Sed		Turb
	Coral morphology	Water	0.87	Turb	0.98	Temp
				Chl a		Turb
				NO_2_		Chl a
				Sed		DO
Fish	Community	Benthic community	0.9	Non-acroporid	0.67	Non-acroporid
				Acroporid		Macroalgae
				Dead coral		Dead coral
		Coral morphology	0.88	Bottlebrush	0.89	Bottlebrush
				Digitate		Digitate
	Feeding guild	Benthic community	0.82	Acroporid	0.81	Soft coral
						Pavement
						Hard coral
						Dead coral
		Coral morphology	0.86	Digitate	0.94	Branching
				Bottlebrush		Bottlebrush
				Submassive		
				Encrusting		

Data is based on the test BioEnv for all sites and those sites located in the outer Thousand Islands.

Fish community taxonomic and feeding guild composition displayed high correlations with both benthic community (p = 0.003 and p = 0.013, respectively) and coral morphology composition (p = 0.002 and p = 0.003, respectively) (Table 5). Fish species richness was linearly correlated with total coral cover (p = 0.03; linear regression). Spatial patterns in both fish community (87% of variability explained) and feeding guild (82% of variability explained) composition were best explained by the total cover of acroporid corals, followed by total cover of non-acroporid and dead corals (Table 5).

**Fig 9:**
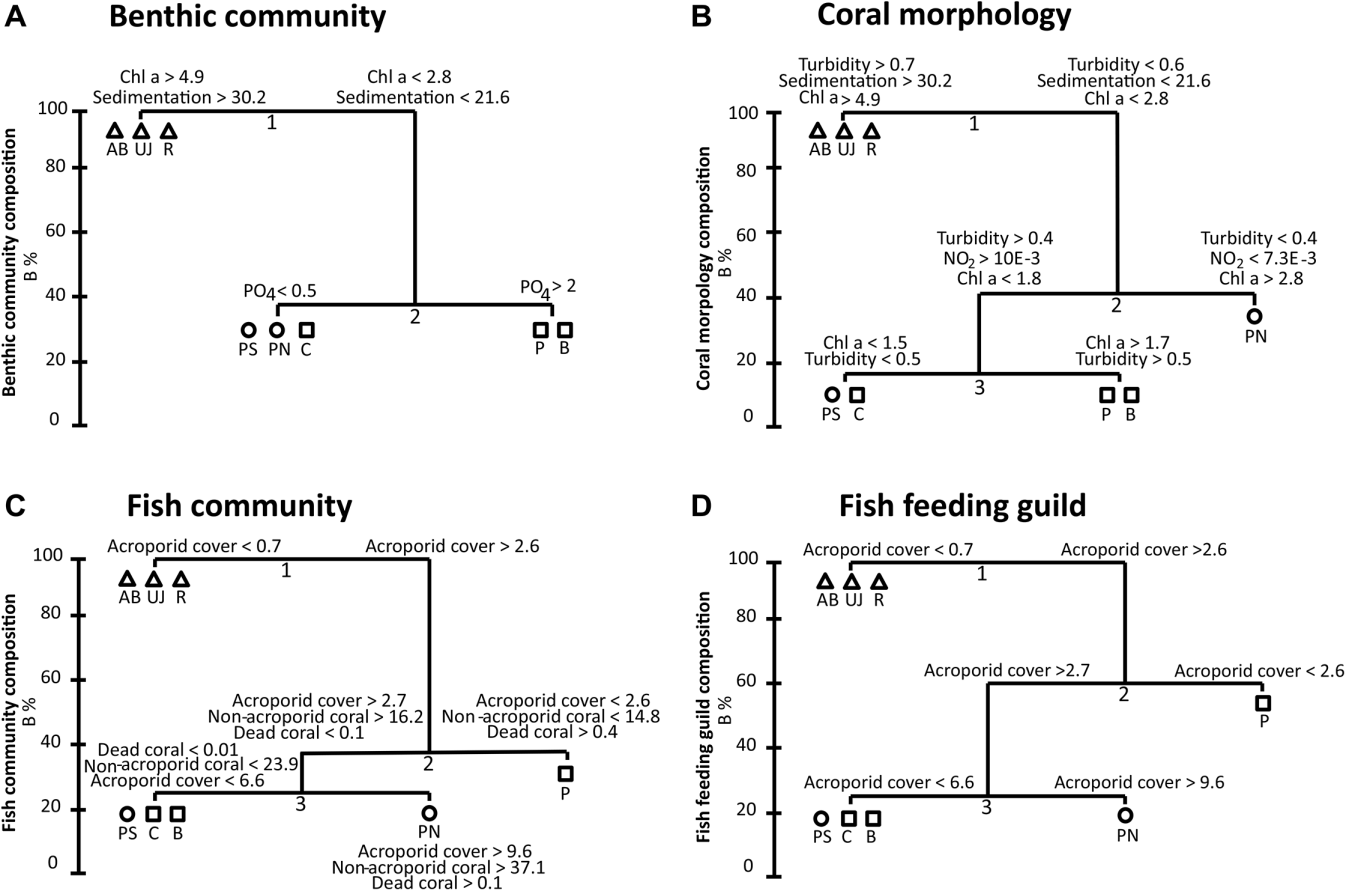
Linkage tree and associated thresholds of proximate drivers that relate to the separation of A) benthic community, B) coral morphology, C) fish community taxonomic and D) fish feeding guild composition. Thresholds at the end of each branch indicate that a left or right path respectively should be followed through the tree. B % is the absolute measure of group differences. Study sites: AB = Ayer Besar, UJ = Untung Jawa, R = Rambut, PS = Pari South, PN = Pari North, P = Panggang, C = Congkak, B = Bira. Zones are indicated by symbols: circle (nearshore), triangle (midshore), square (offshore).

The role of the main drivers for the four different composition groups is visualized in linkage tree graphs (Fig 9) and in all cases shows a clear grouping of sites within JB, but a mix of sites from mid- and offshore reefs due to localized effects of anthropogenic stressors. Both for the fish community and feeding guild composition, Pari North is separated from all other reefs due to highest overall coral cover. Similarly, Panggang is separated due to it having the lowest coral cover overall. In the two benthic composition groups, the sites Panggang and Bira together form one group due to higher PO_4_ levels (benthic community composition) and higher Chl a and turbidity levels (coral morphology composition), compared to the other sites from mid- and offshore reefs.

## Discussion

At the sample depth of 5 m, multiple stressors affected spatial patterns in coral reef communities along the reefs of Jakarta Bay and the Thousand Islands. Using a combination of different models, we found that local and regional factors acted and interacted at different spatial scales, resulting in a mosaic of reef community configurations. Results suggest that for reefs at around 5 m water depth, the direct impact of Jakarta appears to be mainly restricted to inshore reefs within the bay, since both univariate and multivariate analysis of benthic and fish communities showed a clear separation between sites in JB and all sites north of the bay (i.e. the northern Thousand Islands). Reefs north of the bay at this depth range, however, did not follow a gradual spatial pattern with reef condition improving towards north, but rather localized effects of anthropogenic stressors, especially those related to eutrophication, appear to shape the spatial structure of reefs. This has led to the spatial patchwork of differentially degraded reefs that was described by Rachello-Dolmen and Cleary [53].

When the Dutch scientist Umbgrove conducted one of the first marine assessments in Indonesia around the Thousand Islands in 1929, he found a reef system with high species diversity [22]. However, anthropogenic influences were already documented in the early 1900s [54–56], and especially since the 1950s, Jakarta´s rapid population growth has transformed the city into a megacity with more than 14,500 inhabitants km^-2^ in the city area [57], causing the bay to become one of the most polluted in Asia [26]. Here, results confirm that the bay is facing extreme eutrophication coupled with increased primary production and turbidity. PO_4_ levels in the upper layer of JB reached 4 μM/L and DIN levels up to 13 μM/L. Damar [58] reported an annual average of 5.1 μM/L for PO_4_ and 20.1 μM/L for DIN in JB. These extremely high nutrient values are the consequence of massive land runoff, lack of sewage treatment and large-scale agri- and aquaculture. In the wider Jakarta region, about 80% of the wastewater runs directly into the rivers through an open ditch system [59]. At all sites in JB, Chl a levels were between 5 and 15 μg/L, thus far above the Eutrophication Threshold Concentration for Chl a of 0.2–0.3 μg/L [60], indicating high primary productivity. In addition, sites within JB had significantly higher sedimentation rates compared to offshore sites in the Thousand Islands, with up to 30 g m^-2^ d^-1^. Sedimentation is considered to be a principal stress factor for coral reefs [61]; e.g. in Singapore, chronic exposure to high sediment loads is seen as the main stressor affecting corals [62,63]. Similarly, high turbidity levels of 3–5 NTU as found in the bay can reduce light availability and thereby reduce coral photosynthesis and calcification rates [36].

These severe changes in water quality, compared to generally oligotrophic and relatively low turbid waters where coral reefs thrive, can in part explain the massive coral reef degradation. Hard coral cover is now at 2% in the bay, and compared to sites from the northern Thousand Islands, fish abundance was reduced by around 80%. Both uni- and multivariate statistics revealed that at the sites within the bay overall reef condition at shallow depth was far lower compared to those reefs along the Thousand Islands. Even though among all of the measured water factors, only salinity and turbidity in JB were significantly different to offshore sites, multivariate statistics showed that when different aspects of fish and coral community composition were considered, all sites within the bay were clearly separated from the rest. This suggests that even though Jakarta is a megacity with high intensity of urbanisation, industry and shipping, the direct impact on coral reef condition at 5 m depth appears to be restricted to within the bay itself. In addition, the fact that compared to previous studies, where nearshore to offshore gradients in heavy metal pollution, nutrient input and water transparency [13,14], as well as coral cover [15,16] and fish abundance [17], were found, hardly any overall gradual de- or increases from near- to offshore were observed, which shows that the changes in reef condition at 5 m depth and water quality are rather abrupt and not gradual. This may be a reflection of the role of those environmental factors acting at shallower water depth. Both salinity and turbidity are likely to display stronger changes in the surface waters, as plumes of runoff from Jakarta largely float on the surface, particularly beyond areas of mixing in the inner parts of the bay. While a number of factors showed linear gradients, these also displayed a breaking point at either Pari South or Pari North. For example, sedimentation rate and cover of sand gradually increased from Pari South towards offshore, while total coral cover and total fish abundance gradually decreased. Cleary et al. [10] reported a linear increase in shallow-water (< 5 m) coral cover from in- to offshore in 1985, and in 1995 higher cover midshore than offshore. Different patterns to those observed in this study for shallow reefs along the Thousand Islands can be assumed to occur at greater depths, particularly at offshore sites with clearer water conditions, and further research is needed to determine spatial patterns at these depths.

Based on a hydrodynamic model, Koropitan et al. [64] concluded that JB is mainly controlled by water influxes from adjacent marine waters. JB is very shallow with a mean depth of only 15 m, resulting in relatively well-mixed concentrations of for instance nutrients [64]. Even though the south-easterly winds during northwest monsoon could potentially cause polluted water masses from JB to reach the northern Thousand Islands, Koropitan et al. [64] estimated that bottom currents are up to 90% slower than surface flows in JB. The relatively good reef condition at Pari Island, especially when compared to sites further north, may be seen as an indicator that water masses from Jakarta do not considerably affect offshore reefs of the Thousand Islands. Damar [58] and Damar et al. [59] found that the direct impact of estuarine nutrient loads coming from Jakarta is limited to nearshore areas close to river mouths. Similarly, other studies focusing on heavy metals [13,14,65,66] and organic contaminants [67] suggest that these pollutants affect reefs of the northern Thousand Islands far less or almost not at all. Most sources of organic pollutants along the Thousand Islands seem to be the increasing ship traffic and oil spills from oil drilling activities to the northwest of the island chain [68].

Nevertheless, the overall reef condition along the Thousand Islands chain at shallow depths can be considered as being poor since total coral cover in most of the sites was < 25% (threshold based on Gomez and Yap [69]). Values of hard coral cover for near- (2%), mid- (37%) and offshore (22%) reefs were similar to those reported by Cleary et al. [10] for the Thousand Islands chain in 2011. However, the midshore reefs exceed the current average coral cover of 22.1% reported for the Indo-Pacific area [70]. Similarly, fish abundance and species richness in November 2012 were lower at all offshores sites compared to estimates by Madduppa et al. [17]. Fishing pressure currently is concentrated on JB where it is very high, however due to continually decreasing fish yields over the last years [71], fishermen may be forced to move to other fishing grounds further north [72], potentially further threatening the already depleted reef fish resources of the Thousand Islands.

Even though previous studies observed gradients along the Thousand Islands in the past, this was not clearly visible by the time of the present study. Neither was there an indication that the zoning into mid- and offshore reefs as used by Cleary et al. [15] is applicable anymore for reefs at around 5 m water depth. Hardly any factors showed significant differences between mid- and offshore reefs along the Thousand Islands at this depth. Results rather show that certain sites (especially Pari North and Panggang) differed significantly from the other sites, suggesting that the observed high spatial variability in reef condition between sites that are only separated by a few km is most likely due to highly localized effects (which will be discussed below). Such a high spatial variability on a smaller regional scale (e.g. [73]) and local scales of < 20 km (e.g. [74–76]) have been observed in other coral reef regions. This highlights the potential role of stressors, both local and regional, in shaping the structure of benthic communities in coral reefs.

Along the Thousand Islands, the benthic community and coral morphology compositions were significantly related to anthropogenically influenced water parameters. 80% of the variation in benthic community composition along the complete island chain could be linked to factors related to terrestrial run-off and eutrophication, especially NO_3_, sedimentation, turbidity, PO_4_ and Chl a, mirroring the observation from a recent ocean-wide study that local anthropogenic stressors can become the dominant factors shaping benthic reef communities [3]. Eutrophication has been proposed to be the main stress factor for many reefs worldwide. For example, long term monitoring data from the Great Barrier Reef show that the overall reduction in total coral cover by 70% is mainly due to eutrophication [77]. Eutrophication refers to the response of an ecosystem to an increase in nutrient concentrations in the water, which then leads to an increase in algae growth and turbidity [78]. Along the Thousand Islands, overall Chl a levels (mean: 1.7 μg/L) were above the eutrophication threshold levels of 0.2–0.3 μg/L [60] at all sites. While correlative in nature, the results of this study suggest that differences in benthic community and coral morphology composition between sites are caused by the presence of local sources of high nutrient values, as can be seen for the site Panggang, situated relatively in the middle of the island chain and the site Bira in the north. Mean PO_4_ and NH_4_ levels were increased by 60% at Panggang compared to Pari, and at Bira PO_4_ was increased by 30%. These two islands form their own cluster in terms of community composition, with similar nutrient conditions. Benthic community composition was significantly related to increased PO_4_ levels, and coral morphology composition mainly to higher turbidity and Chl a levels. At the site Panggang, the increased nutrient levels can be attributed to the proximity of nearby highly populated islands (Pramuka and Panggang), where sewage is discharged directly to the sea without any prior treatment as commonly practiced on all populated islands along the Thousand Islands chain. However, proximity to populated islands alone is not the only explanatory factor, since Bira Island is not inhabited, and the highest coral cover (both acroporid and non-acroporid cover) was found at Pari North and Pari South close to the populated Pari island complex. This island group displayed relatively low nutrient levels, suggesting that human population density does not always lead to coral decline linked to eutrophication.

However, the observed localized differences in benthic reef condition at 5 m depth along the Thousand Islands are not necessarily caused by local sources of eutrophication alone. Experimental studies are necessary to empirically establish causation, and other confounding factors may play a role as well. Many regional-scale disturbances such as predator and disease outbreaks and bleaching events have been shown to exhibit highly localized effects, leading to high spatial variability on smaller regional scales [73,76]. Cleary et al. [10] suggest that the 2010 bleaching event in the Indo-Pacific region and Indonesia has affected offshore reefs at the Thousand Islands more severely than midshore reefs. The authors reported a dramatic loss of acroporid cover offshore from 36% in 1985 to 5% in 1995. In the present study, cover of rubble increased significantly towards offshore, where it is now at 31%. Although Cleary et al. [10] suggest a tentative recovery for reefs of the Thousand Islands chain, the present observations suggest that coral reef recovery may still be inhibited, at least in shallow areas. Large fields of rubble can shift during storms, which may hinder colonization by coral recruits [79]. Especially at the sites Bira and Panggang, the loss in Acropora cover is severe, with a current cover of < 3%. Similarly, blast fishing was commonly practiced in the 1980s [80].

Overall fish community composition was highly related to the benthic community composition, and almost 90% of variability in fish community composition along the Thousand Islands could be linked to the cover of acroporid, non-acroporid and dead corals. Declines in the abundance and diversity of coral reef fish have been previously linked to an indirect effect of habitat loss [81]. Herbivores play a key role in reef ecosystem function since they actively influence the competition for space between corals and algae [82]. At Pari North and Pari South, a higher herbivore abundance was observed compared to Panggang, where average macroalgae cover was similarly high to that of sites in JB. The disappearance of herbivores as a top-down factor has been suggested to cause shifts of coral reefs towards macroalgae-dominated conditions, rather than bottom-up factors such as eutrophication [83]. Such shifts have been documented from locations around the world, but to date appear most common in the Caribbean [84].

High spatial variability in reef condition on a regional scale, as observed for the reefs of the Thousand Islands, has to be considered in future conservation and management plans. For the Thousand Islands this specifically means that the organization of the Thousand Islands National Park has to be re-evaluated, since the regulations as well as the boundaries of the park have not undergone any reformation for almost three decades [66], to account for demographic, economic and environmental changes. For example, there are four different government agencies involved in decision making processes regarding the National Park organization, which work independently and consequently can slow down overall progress [66]. Localized effects have to be incorporated into management plans, and marine spatial planning that explicitly accounts for the different spatial extent of stressors as an alternative to conventional management could be a suitable approach. Marine spatial planning tries to consider the needs of all stakeholders involved in a certain system by assigning defined zones, which could be based on their distance to an urban centre to reflect differences in impacts and uses. In the present case, zoning should not only include distance from Jakarta, but consider island-specific conditions that may fall outside of larger-scale trends. For example, an approach could be to administer each populated island separately rather than grouping several populated islands into an administrative zone, as is currently the case for the Thousand Islands. Each zone may then be assigned to certain activities, for example port and shipping closer to the city centre, followed by fishing activities, then tourism and furthest away marine reserves [5]. As such a zonation roughly reflects the current spatial arrangement of activities in the Thousand Islands, incorporating them into a dedicated Marine Spatial Planning framework should be feasible. However, the specific situation in the area necessitates a few location-specific adjustments. For example, the extreme levels of pollution in the inner Jakarta Bay have caused significant heavy metal contamination in green mussels [85], which suggests that the intense aquaculture of green mussels currently practiced there is not sustainable in terms of hazards to human health (as discussed in the news: [86]) and that further research is urgently needed. Similarly, the Thousand Islands National Park currently has conservation, residential (including fisheries) and tourism zones, however results from the present study suggest that reefs in conservation zones (Bira Island) may not necessarily have higher coral cover than reefs in areas with less protection (Congkak and Panggang) or even reefs outside of the boundaries of the National Park (Pari Island), and that this reality should be reflected in a revised zonation plan.

The current study indicated the potential for multiple stressors to interact to varying degrees along the island chain. Localized stressors appeared to shape the spatial structure of reefs rather than regional stressors. Nevertheless, it is still very difficult to study the effects of pollution on coral reefs. Cumulative effects of multiple stressors on ecological communities are barely understood, and the response of organisms and ecosystems to a suite of stressors is still not clear due to varying tolerance thresholds of the different species and complex interactions between organisms and stressors. The present study yielded correlative results, and although the observed correlations were quite strong, these should be followed by manipulative experiments to establish causal agents among the identified stressors and their interactions. In studies of interactive effects of stressors, pollution remains one of the least understood factors [87]. Furthermore, Crain et al. [88] propose that synergies may be quite common in nature, complicating the prediction of interactive effects. In addition, the effects of pollution at regional scales (compared to a single local reef) are harder to distinguish due to confounding stressors such as bleaching events, storms, fishing pressure and coral diseases. At the same time, the natural variation of reefs along water quality gradients in the absence of well-defined pollution sources has to be considered, i.e. reefs close to the main coastline are often characterized by different nutrient loads, turbidity, wave exposure and current conditions compared to reefs with oceanic conditions [89].

Monitoring key biological and environmental parameters continuously over several years and across seasons is key to establish successful management and conservation plans. Any conservation and management plan, however, will only be successful if pollution in Jakarta is reduced, e.g. by implementing sewage treatment and waste disposal plans as well as reducing air pollution. Tackling these massive problems will require all stakeholders to work together, a pro-active government, and a reduction in corruption [26,90]. In addition, marine spatial planning that is adjusted to local conditions and takes into consideration the different spatial scales on which stressors and resource uses interact with reef communities is required to adequately address the current situation of Jakarta Bay and the Thousand Islands. While these are considerable challenges, complacency is not an option. When considering the importance of coral reefs for the livelihoods of millions of people in developing countries, including large parts of the population in large cities, the need for coral reef conservation in the vicinity of large cities such as Jakarta is obvious and repercussions of degrading habitat reef conditions are likely to be far reaching.

## Supporting Information

S1 Fig r-K-S ternary plot for benthic community composition along the Thousand Islands. R: disturbance-adapted ruderal corals (acroporid corals); K: competition-adapted corals (branching non-acroporid corals and foliose corals); S: stress-tolerating corals (massive and submassive corals).
(EPS)

S2 Fig Visualization of composition groups based on a priori defined groups, i.e. zones. Canonical analysis of principal coordinates (CAP) was used for (A) benthic community, (B) coral morphology, (C) fish community and (C) fish feeding guild composition.
(EPS)

S3 Fig Total fish abundance (A) and species richness (B) for sites in Jakarta Bay (JB) and Thousand Islands. Mean values given for the families Labridae (excluding Scarinae), Pomacentridae, Nemipteridae, labrid Scarinae, Serrranidae and Caesionidae, and Others.
(EPS)

S1 Table Fish survey 2012. Average abundance of observed fish species per zone and assigned feeding guild for each species after FishBase (Froese and Pauly 2000): OV = omnivore, OCV = obligate corallivore, HV = herbivore, PV = planktivore, CV = carnivore, OVI = omnivore/invertivore.
(DOCX)

S2 Table Multivariate analysis of composition groups based on zonation. The Permanova test and subsequent pairwise testing of zones was used.
(DOCX)

S3 Table Coral morphology categories for Indonesian coral reefs used to classify rks-groups (Edinger and Risk 2000). (DOCX)
