# Supplementary material for: Local and Regional Impacts of Pollution on Coral Reefs along the Thousand Islands North of the Megacity Jakarta, Indonesia
Source: PLoS One. 2015 Sep 17;10(9):e0138271. doi: 10.1371/journal.pone.0138271 (PMC4574762; doi:10.1371/journal.pone.0138271)
Supplement: S1 Table — Average abundance of observed fish species per zone and assigned feeding guild for each species after FishBase (Froese and Pauly 2000): OV = omnivore, OCV = obligate corallivore, HV = herbivore, PV = planktivore, CV = carnivore, OVI = omnivore/invertivore. (DOCX) [file pone.0138271.s004.docx]

|  |  |  | **Feeding guild** | **Abundance per zone** | | | **Total abundance** |
| --- | --- | --- | --- | --- | --- | --- | --- |
| **No.** | **Family** | **Species** |  | **1** | **2** | **3** |  |
| 1 | **Acanthuridae** | *Acanthurus tristis* | PV |  | 2 |  | 2 |
| 2 |  | *Ctenochaetus striatus* | OV |  | 2 |  | 2 |
| 3 | **Apogonidae** | [*Ostorhinchus compressus*](http://fishbase.org/summary/SpeciesSummary.php?id=5769) | OV | 9 | 38 | 23 | 70 |
| 4 |  | *Cheilodipterus intermedius* | OV |  |  | 12 | 12 |
| 5 |  | *Cheilodipterus quinquelineatus* | OVI | 6 |  |  | 6 |
| 6 |  | *Fibramia thermalis* | OV | 4 |  |  | 4 |
| 7 | **Aulostomidae** | *Aulostomus chinensis* | CV |  | 1 | 1 | 2 |
| 8 | **Caesionidae** | *Caesio cuning* | PV | 31 | 24 | 34 | 89 |
| 9 | **Centriscidae** | *Aeoliscus strigatus* | PV | 24 |  |  | 24 |
| 10 | **Chaetodontidae** | *Chaetodon octofasciatus* | OCV | 3 | 5 | 4 | 12 |
| 11 |  | *Chelmon rostratus* | OCV | 1 |  | 2 | 3 |
| 12 |  | *Heniochus pleurotaenia* | OCV |  | 3 | 2 | 5 |
| 13 | **Diodontidae** | *Diodon hystrix* | OVI |  | 1 |  | 1 |
| 14 | **Gramistidae** | *Diploprion bifasciatum* | CV | 3 |  |  | 3 |
| 15 | **Haemulidae** | *Plectorhinchus lineatus* |  | 1 |  |  | 1 |
| 16 | **Holocentridae** | *Sargocentron rubrum* | CV | 5 |  |  | 5 |
| 17 | **Labridae** | *Bodianus mesothorax* |  |  | 3 |  | 3 |
| 18 |  | *Cheilinus fasciatus* | OVI |  | 4 | 5 | 9 |
| 19 |  | *Cheilinus undulatus* | CV |  |  | 2 | 2 |
| 20 |  | *Choerodon anchorago* | OVI | 5 | 3 | 3 | 11 |
| 21 |  | *Cirrhilabrus cyanopleura* | PV | 20 | 96 | 57 | 173 |
| 22 |  | *Halichoeres binotopsis* | CV | 4 |  |  | 4 |
| 23 |  | *Halichoeres chloropterus* | CV | 3 | 5 | 4 | 12 |
| 24 |  | [*Halichoeres melanurus*](http://fishbase.org/summary/SpeciesSummary.php?id=4858) | CV | 3 | 5 | 3 | 11 |
| 25 |  | *Halichoeres hortulanus* | OVI |  | 5 | 2 | 7 |
| 26 |  | *Halichoeres melanurus* | OV | 2 |  |  | 2 |
| 27 |  | *Hemigymnus fasciatus* | OVI |  |  | 3 | 3 |
| 28 |  | *Hemigymnus melapterus* | OVI |  | 4 | 3 | 7 |
| 30 |  | *Labroides bicolor* | OVI |  | 1 |  | 1 |
| 31 |  | *Labroides dimidiatus* | OVI | 2 | 3 | 3 | 8 |
| 32 |  | *Stethojulis interrupta* |  |  |  | 1 | 1 |
| 33 |  | *Stethojulis trilineata* | OVI | 3 | 2 |  | 5 |
| 34 |  | *Thalassoma lunare* | OVI |  | 10 | 6 | 16 |
| 35 | **Lethrinidae** | *Lethrinus harak* | CV | 2 |  |  | 2 |
| 36 | **Lutjanidae** | *Lutjanus carponotatus* | CV |  |  | 2 | 2 |
| 37 |  | *Lutjanus decussatus* | CV | 1 | 3 |  | 4 |
| 38 | **Mullidae** | *Upeneus tragula* |  | 2 |  |  | 2 |
| 39 | **Nemipteridae** | *Pentapodus setosus* | OVI | 6 |  |  | 6 |
| 40 |  | *Scolopsis aurata* | OVI |  |  | 3 | 3 |
| 41 |  | *Scolopsis bilineata* | CV |  | 6 | 5 | 11 |
| 42 |  | *Scolopsis ciliata* | CV | 6 |  |  | 6 |
| 43 |  | *Scolopsis margaritifera* | CV |  | 4 | 3 | 7 |
| 44 |  | *Scolopsis monogramma* | OVI | 5 | 4 |  | 9 |
| 45 |  | *Scolopsis vosmeri* | OV | 2 | 3 |  | 5 |
| 46 | **Ostraciidae** | *Ostracion cubicus* | OV |  | 1 | 1 | 2 |
| 47 | **Phemperidae** | *Pempheris vanicolensis* | PV | 8 | 22 |  | 30 |
| 48 | **Pomacanthidae** | *Chaetodontoplus mesoleucus* | OV |  | 3 | 2 | 5 |
| 49 |  | *Pomacanthus sexstriatus* | HV |  | 2 | 2 | 4 |
| 50 | **Pomacentridae** | *Abudefduf bengalensis* | OV | 4 | 7 |  | 11 |
| 51 |  | *Abudefduf sexfasciatus* | OV | 3 | 32 | 12 | 47 |
| 52 |  | *Abudefduf vaigiensis* | OV |  | 22 | 11 | 33 |
| 53 |  | *Amblyglyphidodon curacao* | OV | 3 | 33 | 37 | 73 |
| 54 |  | *Amblyglyphidodon leucogaster* | OV |  | 18 | 9 | 27 |
| 55 |  | *Amphiprion ocellaris* | OV | 4 |  |  | 4 |
| 56 |  | *Chromis viridis* | OV |  | 47 | 30 | 77 |
| 57 |  | *Chrysiptera hemicyanea* | OV | 10 |  |  | 10 |
| 58 |  | *Dischistodus prosopotaenia* | HV |  | 5 | 5 | 10 |
| 59 |  | *Neoglyphidodon crossi* | OV |  | 10 |  | 10 |
| 60 |  | *Neoglyphidodon melas* | OV |  | 7 | 7 | 14 |
| 61 |  | *Neoglyphidodon nigroris* | OV |  | 7 | 11 | 18 |
| 62 |  | *Neopomacentrus anabatoides* | PV | 12 | 21 |  | 33 |
| 63 |  | *Neopomacentrus azysron* | OV |  | 8 | 21 | 29 |
| 64 |  | *Neopomacentrus cyanomos* | CV | 5 | 20 |  | 25 |
| 65 |  | *Pomacentrus alexanderae* | OV | 5 | 33.5 | 48 | 86.5 |
| 66 |  | *Pomacentrus amboinensis* | HV | 7 | 12 | 9 | 28 |
| 67 |  | *Pomacentrus armillatus* | HV | 8 | 12 | 8 | 28 |
| 68 |  | *Pomacentrus bankanensis* | OV |  | 8 | 33 | 41 |
| 69 |  | *Pomacentrus burroughi* | HV |  |  | 6 | 6 |
| 70 |  | *Pomacentrus chrysurus* | HV | 5 |  |  | 5 |
| 71 |  | *Pomacentrus cuneatus* | OV | 8 |  |  | 8 |
| 72 |  | *Pomacentrus javanicus* | OV | 11 |  |  | 11 |
| 73 |  | *Pomacentrus lepidogenys* | PV |  |  | 70 | 70 |
| 74 |  | *Pomacentrus littoralis* | OV | 7 |  |  | 7 |
| 75 |  | *Pomacentrus moluccensis* | HV |  | 30 | 19 | 49 |
| 76 |  | *Pomacentrus nigromarginatus* | PV |  | 8 |  | 8 |
| 77 |  | *Pomacentrus philippinus* | OV |  |  | 18 | 18 |
| 78 |  | *Pomacentrus saksonoi* |  |  |  | 8 | 8 |
| 79 |  | *Pomacentrus smithi* | OV |  | 38 | 57 | 95 |
| 80 |  | *Premnas biaculeatus* | OV | 3 |  | 2 | 5 |
| 81 | **Scaridae** | *Cetoscarus bicolor* | HV |  | 2 |  | 2 |
| 82 |  | *Chlorurus bleekeri* | HV |  | 4 |  | 4 |
| 83 |  | *Chlorurus bowersi* | OV |  |  | 2 | 2 |
| 84 |  | *Chlorurus sordidus* | OV |  | 4 | 6 | 10 |
| 85 |  | *Scarus ghobban* | HV |  | 2 | 2 | 4 |
| 86 |  | *Scarus niger* | HV |  | 4 | 4 | 8 |
| 87 |  | *Scarus quoyi* | HV |  | 1 |  | 1 |
| 88 | **Serranidae** | *Cephalopholis boenak* | CV | 2 | 2 | 3 | 7 |
| 89 |  | *Cephalopholis microprion* | CV |  |  | 3 | 3 |
| 90 |  | *Epinephelus fasciatus* | CV |  |  | 1 | 1 |
| 91 | **Siganidae** | *Siganus virgatus* | HV |  | 3 |  | 3 |
| 92 |  | *Siganus rivulatus* | HV |  | 3 | 4 | 7 |
