# Supplementary material for: Local and Regional Impacts of Pollution on Coral Reefs along the Thousand Islands North of the Megacity Jakarta, Indonesia
Source: PLoS One. 2015 Sep 17;10(9):e0138271. doi: 10.1371/journal.pone.0138271 (PMC4574762; doi:10.1371/journal.pone.0138271)
Supplement: S2 Table — The Permanova test and subsequent pairwise testing of zones was used. (DOCX) [file pone.0138271.s005.docx]

| **Group** | **Composition** | **Test pairs zones** | **p-value** |
| --- | --- | --- | --- |
| Fish | Community | global | **0.002** |
|  |  | 1 vs. 2 | 0.101 |
|  |  | 1 vs. 3 | 0.079 |
|  |  | 2 v.s 3 | 0.105 |
|  | Feeding guild | global | **0.03** |
|  |  | 1 vs. 2 | 0.089 |
|  |  | 1 vs. 3 | 0.116 |
|  |  | 2 v.s 3 | 0.477 |
| Benthic | Community | global | **0.017** |
|  |  | 1 vs. 2 | 0.104 |
|  |  | 1 vs. 3 | 0.091 |
|  |  | 2 v.s 3 | 0.704 |
|  | Coral morphology | global | **0.008** |
|  |  | 1 vs. 2 | 0.084 |
|  |  | 1 vs. 3 | 0.11 |
|  |  | 2 v.s 3 | 0.194 |
| Water |  | global | **0.008** |
|  |  | 1 vs. 2 | 0.092 |
|  |  | 1 vs. 3 | 0.105 |
|  |  | 2 v.s 3 | 0.101 |
