# Supplementary material for: Local and Regional Impacts of Pollution on Coral Reefs along the Thousand Islands North of the Megacity Jakarta, Indonesia
Source: PLoS One. 2015 Sep 17;10(9):e0138271. doi: 10.1371/journal.pone.0138271 (PMC4574762; doi:10.1371/journal.pone.0138271)
Supplement: S3 Table — (DOCX) [file pone.0138271.s006.docx]

| **Morphology** | **Description** | **rKS group** |
| --- | --- | --- |
| Acropora, branching | Staghorn corals, long thin branches | r |
| Acropora, bottlebrush | Mainly *A. echinata* group | r |
| Acropora, corymbose | Stout branches, low bushy shape | r |
| Acropora, digitate | Digitate, stubby, mainly *A. humilis* group | r |
| Acropora, tabular | Tables, mainly *A. hyacinthus* group | r |
| Acropora, submassive | Columns + blades, very stout, mainly *A. palifera and A. cuneata* | r |
| Branching coral | Branching non-Acropora corals; especially *Porites cylindrica*, some other spp. | K |
| Encrusting coral | Low relief, often small colonies | K |
| Massive-platy coral (Lobophyllia spp.) | Plate-like corals forming large massive colonies, especially Euphyllia, | S |
| Massive coral | Massive or dome-like corals of all sizes. | S |
| Foliose coral | Foliose, either horizontal or vertical, non-Acropora, especially Montipora, Echinopora | K |
| Tabular coral (non-Acropora) | Tabular non-Acropora, esp. Montipora | r |
| Submassive coral columnar | Multilobate or ``lumpy'' corals, sometimes columnar or mixed massive-columnar, especially Goniopora, Galaxea | S |
| Mushroom coral | Free-living fungiid corals | K |
| Millepora | Various species of Millepora. (hydrocoral) | r |
| Heliopora | Blue coral (a hydrocoral) | r |
